# Complete genome sequence of *Sporosarcina pasteurii* type strain DSM33

**DOI:** 10.1128/mra.00843-24

**Published:** 2024-11-20

**Authors:** Matthew J. Tuttle, Blake W. Stamps, John L. Chodkowski, Marco E. Mechan-Llontop, Ashley Shade, Chia-Suei Hung, Maneesh K. Gupta, Michael S. Carter

**Affiliations:** 1 Materials and Manufacturing Directorate, Air Force Research Lab, Wright-Patterson Air Force Base, Dayton, Ohio, USA; 2 Biological and Nanoscale Technologies Division, UES Inc., a BlueHalo Company, Dayton, Ohio, USA; 3 Department of Microbiology, Immunology and Genetics, Michigan State University, East Lansing, Michigan, USA; 4 Universite Claude Bernard Lyon, Lyon, France; DOE Joint Genome Institute, Berkeley, California, USA

**Keywords:** MICP, biocement, *Sporosarcina pasteurii*, urease, biomineralization, biotechnology, soil stabilization, environmental engineering

## Abstract

Here, we present the complete genome sequence of *Sporosarcina pasteurii* type strain DSM33, a model organism for microbially induced calcium carbonate precipitation. This genome consists of a single 3.3-Mb chromosome and is an improvement upon draft *S. pasteurii* genome sequences currently available in public databases.

## ANNOUNCEMENT

**Fig 1:**
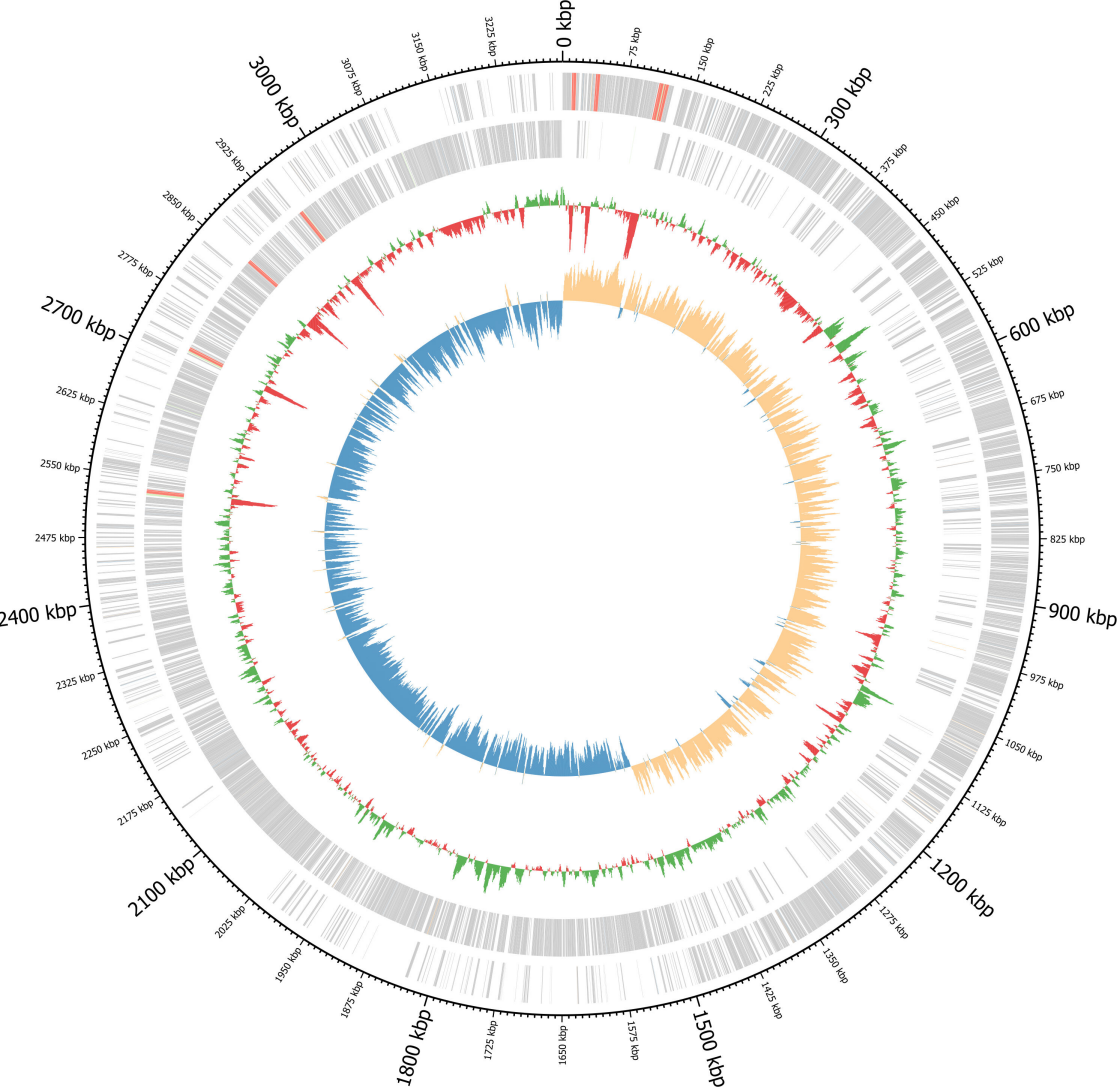
Circular genome map of the complete *S. pasteurii* DSM33 genome. Moving from the outer circle inward, the five circular tracks represent (i) sequence location in kbp, (ii) forward-strand genome features, (iii) reverse-strand genome features, (iv) GC content relative to average, and (v) GC skew. Forward- and reverse-strand features include CDSs (gray), tRNA/tRNAs (green), rRNAs (red), ncRNAs (orange), and ncRNA regions (blue). This plot was generated by Bakta (7) using Circos (8).

*Sporosarcina pasteurii* is a Gram-positive endospore-forming alkaliphilic bacterium first isolated in 1889 (1, 2). Most strains produce high concentrations of urease (3). *S. pasteurii* DSM33 (also known as ATCC 11859) and other *S. pasteurii* strains have emerged as models for the production of biocement via microbially induced calcium carbonate precipitation, given their high levels of urease activity (4–6). *S. pasteurii* DSM33 is the most commonly used strain, so, here, we sequenced and assembled its complete genome (Fig. 1).

*S. pasteurii* DSM33 was obtained from the German Collection of Microorganisms and Cell Cultures (Leibniz Institute DSMZ). DSM33 was grown in brain–heart infusion broth (MilliporeSigma) amended with 2% (wt/vol) urea (MilliporeSigma) for 18 hours at 30 ˚C with 250 rpm shaking prior to DNA extraction. For Nanopore sequencing (Oxford Nanopore Technologies), DNA was isolated via phenol–chloroform extraction (9). Nanopore sequencing libraries were prepared using a Ligation sequencing kit (SQK-LSK109; Oxford Nanopore Technologies). Libraries were sequenced using a FLO-MIN111 flow cell on a MinION sequencer. Base calling was performed using Guppy (high-accuracy model; v5.0.16; Oxford Nanopore Technologies), reads were quality-controlled using MinIONQC (v1.4.1; Q ≥ 10) (10), and adapters trimmed using Porechop (v0.2.4) (11). A total of 76,442 raw reads were obtained with an N50 of 22,709 bp. For Illumina sequencing, DNA was extracted from an independent sample of DSM33 using a DNeasy Blood & Tissue Kit (QIAGEN) following the manufacturer’s protocol for Gram-positive bacteria. Illumina libraries were prepared using a SeqWell purePlex DNA Library Prep Kit, as per the manufacturer’s directions. After preparation, the library pool quality was assessed using a QuBit BR assay (ThermoFisher Scientific) for concentration and insert size using an Agilent Tapestation D5000 kit. The sample was then denatured, diluted to a load concentration of 10 pM, and 250-bp paired-end reads were sequenced using an Illumina MiSeq 500 cycle V2 kit, yielding 6.27 million raw reads. Illumina reads were trimmed for quality, adapters, and length (minimum length 150 bp; minimum Q score 30) using FastP. DNA assembly of Nanopore reads was first performed using the Trycycler pipeline (v0.5.3) (12) using default parameters with Flye (v2.9.1-b1780) (13), Raven (v1.8.1) (14), and miniasm (v0.3-r179) (15) as the assemblers. Post consensus, in Trycycler, the assembly was polished with the Illumina reads using Polypolish (v0.5.0) and POLCA (within mascara v4.1.0). Annotations were generated using the NCBI Prokaryotic Genome Annotation Pipeline (PGAP; v6.7) (16).

The final genome assembly comprised a complete genome sequence consisting of a single circular chromosome 3.3 Mb in size with a G + C content of 39.2%. Average nanopore read coverage was 109-fold. Trycycler assembly (12) circularized the genome, which was rotated by the assembler to place *dnaA* at the start of the contig. Genome annotation resulted in 3,115 protein-coding sequences (CDSs), eight rRNA operons, 69 tRNAs, and nine ncRNAs.

## Data Availability

The genome sequence of *S. pasteurii* DSM33 is available in GenBank under accession number CP160452. Raw sequence reads are available under the Sequence Read Archive (SRA) accession numbers SRR28831121 (Illumina) and SRR28967830 (Oxford Nanopore Technologies).
